# Perceived Stress among Caregivers of Children with Autism Spectrum Disorder: A State-Wide Study

**DOI:** 10.3390/ijerph16081468

**Published:** 2019-04-25

**Authors:** Nik Aida Nik Adib, Mohd Ismail Ibrahim, Azriani Ab Rahman, Raishan Shafini Bakar, Nor Azni Yahaya, Suria Hussin, Wan Nor Arifin Wan Mansor

**Affiliations:** 1 Department of Community Medicine, School of Medical Sciences, Universiti Sains Malaysia, 16150 Kota Bharu, Kelantan, Malaysia; eyedasyukran@gmail.com (N.A.N.A.); azriani@usm.my (A.A.R.); 2 Department of Psychiatry, School of Medical Sciences, Hospital Universiti Sains Malaysia, 16150 Kota Bharu, Kelantan, Malaysia; raishanshafini@usm.my; 3 Department of Pediatrics, Hospital Raja Perempuan Zainab II, 15200 Kota Bharu, Kelantan, Malaysia; drnorazni@moh.gov.my; 4 Department of Psychiatry, Hospital Raja Perempuan Zainab II, 15200 Kota Bharu, Kelantan, Malaysia; drsuria@moh.gov.my; 5 Unit of Biostatistics and Research Methodology, School of Medical Sciences, Universiti Sains Malaysia, 16150 Kota Bharu, Kelantan, Malaysia; wnarifin@usm.my

**Keywords:** perceived stress, caregivers, autism spectrum disorder

## Abstract

Background: Caregivers of children with autism spectrum disorder (ASD) experience increased stress and more significant negative caregiving consequences than those with typically developing children. There is a lack of studies specifically focusing on stress among caregivers with ASD children in Asian countries. The current study examines levels of perceived stress and factors associated with it among caregivers in Kelantan, Malaysia. Methods: In a cross-sectional study, the Malay version of the Perceived Stress Scale (PSS) was administered to 227 caregivers of children with ASD. The caregivers were recruited from ASD databases in four tertiary hospitals in Kelantan and a meeting was set up during the child’s follow-up in the clinic. Multiple linear regression analyses were applied to determine the predictors of perceived stress. Results: The mean total perceived stress score was 20.84 (4.72). This was considered higher than average. Higher perceived stress was significantly predicted among caregivers who live far from the health institution, caregivers who do not own transportation to bring the child to the treatment center, and caregivers who have an ASD child with a learning disability. Conclusion: Caregivers of an ASD child perceived significant stress while taking care of their children. Institutions should alleviate the factors that were predicted to increase the caregivers’ perceived stress to improve the quality of the lives of children and ASD families as a whole.

## 1. Introduction

Autism spectrum disorder (ASD) is a neurodevelopmental disorder characterized by deficits in social communication, social interaction, and restricted repetitive patterns of behavior, interests, and activities beginning in childhood. It is often comorbid with other conditions, such as intellectual impairment, disruptive behaviors, attention difficulties, aggression, poor eating, sleep problems, epilepsy, gastrointestinal problems, and motor coordination [1,2]. 

The prevalence of ASD worldwide has risen steadily. The global prevalence of ASD has increased up to thirtyfold since the earliest epidemiologic studies began tracking it in the late 1960s and early 1970s [3]. The reasons for the dramatic increase in ASD prevalence are currently being debated in the literature, which postulates several causes, including a rising awareness among caregivers, improvements in the diagnostic criteria based on the Diagnostic and Statistical Manual of Mental Disorders, 5th edition (DSM-5), and advances in services that drive accurate diagnoses [4,5]. 

According to a recent report by Malaysia’s Social Welfare Department, 420,201 children and adults were registered to have some kind of disability in Malaysia. Learning disability (LD) comprised nearly 40% of the total number of those registered. Data from December 2018 show 39,321 people registered as disabled in Kelantan and 13,642 of those were registered with an LD [6]. 

ASD was grouped under LD and ranked as the first type of disability in Malaysia [7]. The prevalence of children with an LD is hard to estimate and is usually underestimated [8]. Nevertheless, there has been no official registry for ASD children in Malaysia. 

A study conducted by the Ministry of Health among children aged 18–26 months revealed that one in every 625 Malay children was diagnosed to have ASD. Regardless of the number, it seems that many ASD cases remain unrecognized. Medical and educational practitioners have been seeing a rise in the number of children with speech delay and communication difficulties who might require further assessment [9]. Thus, it seems that a true prevalence rate of ASD in Malaysia is probably much higher.

Higher levels of stress and other negative psychological outcomes are a few of the challenges faced by caregivers of children with developmental disabilities. Caregiving to a child with ASD may pose additional stressors related to the child’s inability to communicate well and unpredictable behaviors, social isolation, difficulties in self-care, and lack of community understanding. Several studies have reported evidence of an increase in psychological distress, including depression, anxiety, decreased family cohesion, increased somatic complaints, and burnout among caregivers of children with ASD as compared to caregivers of children with other developmental disabilities [10,11].

The economic impact on families raising children with ASD is between three and five dollars more per day than normal-growing children. This figure increases significantly when the ASD child also has severe cognitive impairment. Even when federal and state programs are in place to provide support to reduce the financial burden on families, caring for ASD children significantly affects the parents’ and families’ time for either work or leisure [12,13]. Compared to caregivers of a normal growing child, caregivers of children with ASD are found to have restricted leisure activities even at home because they need to spend more time taking care of their disabled child [14]. This situation may lead to the caregivers being unable to find a proper job, may add increased financial stressors on the families, and may diminish parents’ resources for both social and emotional support. 

Stressful situations experienced by caregivers of children with ASD are considered to be crucial in determining quality of life and family functioning. A study conducted by Cramm and Nieboer [15] demonstrated that parental stress is a strong predictor of caregivers’ psychological well-being and usually results in decisions to place children with intellectual disabilities in the care of others. 

Despite the extensive knowledge of stress in parents of children with LDs, research specifically focused on ASD remains understudied in developing countries like Malaysia. Therefore, it is very important to recognize stress among local caregivers as it contributes to not only their own psychological well-being but also to their children’s development and management. Identification of caregivers’ stress is also required to improve informational resources and support services that meet their needs and desires and that indirectly reduces caregivers’ stressors.

The northeast state of Peninsular Malaysia, notably Kelantan, is recorded as relatively poor with a mean monthly income of USD 666 with a Gross Domestic Product (GDP) of 5.0 in 2017. This is comparatively higher than a few other states in Malaysia [16]. The overall prevalence of adequate health literacy among adults aged 18 years and above was 6.6% (95% Confidence interval (CI): 5.6–7.7) in Malaysia with no differences between gender, based on self-administered questionnaires using the Newest Vital Sign tool [17]. Therefore, management for specific healthcare problems such as ASD need improvement because of limited resources in Kelantan. Caregivers are also exposed to socio-structural limitations leading to psychological distress. Thus, the aim of the current study was to determine the level of perceived stress among caregivers of children with ASD and factors associated with it in Kelantan.

## 2. Materials and Methods 

### 2.1. Study Setting and Participants 

This was a cross-sectional study conducted in four tertiary healthcare centers in Kelantan: Hospital Universiti Sains Malaysia, Hospital Raja Perempuan Zainab II, Hospital Kuala Krai, and Hospital Tanah Merah. The study, which began in February 2018 and ran for two months, involved caregivers of a child with ASD who had received treatment and follow-up from the healthcare centers. A stratified, random sampling method was used to select the respondents from the identified list of children with ASD that came for follow-up. All eligible samples were included in the study. Since we used a self-administered questionnaire, those primary caregivers who were illiterate were excluded from the study to avoid bias and to standardize the method of data collection. Also excluded were caregivers known to have an underlying psychiatric problem and children for whom an ASD diagnosis was not yet confirmed during the study period. In the current study, a person was defined as the primary caregiver if they were primarily responsible for the development of the child and most involved in taking care of him or her, whether during hospitalization, specialist consultation, or any clinical intervention. The primary caregivers who consented to participate in the study were given a Malay version of the Perceived Stress Scale (PSS-M) questionnaire to be filled out together with caregiver characteristics. The self-administered questionnaire was performed in a clinical setting at an occupational therapy clinic at one of the tertiary healthcare centers. 

### 2.2. Perceived Stress Scale-10 Items (PSS-10)

The PSS-10, developed by Cohen, Kamarck and Mermelstein [18], is the most widely used psychological instrument for measuring the perception of stress. The questions in the PSS-10 ask about feelings and thoughts during the last month. The 10 items of PSS-10 were rated on a five-point Likert scale based on the frequency of the stressful event experienced by the participant (0 = never, 1 = almost never, 2 = sometimes, 3 = fairly often, 4 = very often). In determining the total PSS score, only the scores for questions 4, 5, 7, and 8 were reversed as follows: 0 = 4, 1 = 3, 2 = 2, 3 = 1, and 4 = 0. The rest of the items remained the same. Then, scores for each item were added to get a total PSS score. The higher the score, the higher the stress perceived by the participant. The Malay version of PSS-10 was translated and validated by Al-Dubai and Alshagga [19] among 242 Bachelor of Medical Science students in a private university in Malaysia. The translation has a good internal consistency reliability with a Cronbach’s alpha coefficient of 0.78 for the overall scale. It also has good factor loading values for all items (0.67 to 0.84) [19]. The Malay version of the PSS-10 was also translated and validated by Mazlan and Ahmad [20]. The Cronbach’s alpha coefficient was found acceptable (α = 0.64) with a high total, test-retest reliability (*R* = 0.72). 

### 2.3. Statistical Analysis

In the current study, all data were entered using Statistical Package for the Social Sciences (SPSS) software version 24.0 [21]. Data were checked and cleaned. Preliminary data screening was done for missing values or possible wrong data entry. Correction for any wrong data entry or completion of missing values was done by re-scrutinizing information recorded in the pro forma. Missing data for specific individual items of the study were excluded from analysis. Exploration also involved examination of data redundancy. Redundant data were deleted accordingly. Data analysis was conducted using IBM SPSS 24.0 to determine the level of caregivers’ perceived stress [21]. Descriptive analysis was used to describe socio-demographic characteristics of the caregivers, socio-demographic characteristics of the children with ASD, and perceived stress. Results were presented as mean and standard deviation (SD) for normally distributed data. Median and inter-quartile range were used to describe skewed data. For categorical data, results were presented as frequency and percentage (%). A regression analysis was run using R version 3.3 (R Foundation for Statistical Computing, Vienna, Austria, 2013) to identify the predictors of perceived stress among caregivers with ASD children.

### 2.4. Ethical Considerations

The study was ethically approved by the Human Research Ethics Committee of Universiti Sains Malaysia (JEPeM Code: USM/JEPeM/17110600) and the National Medical Research Register (NMMR) Malaysia (NMMR-17-2732-38655). The confidentiality of the data was strictly protected. All reporting and publication were carried out in complete anonymity with no respondents named. 

## 3. Results

### 3.1. Socio-Demographic Characteristics of the Caregivers

The socio-demographic features of the caregivers are listed in Table 1. Their mean standard deviation (SD) age was 38.91 (8.26) years. The majority were female (74.9%) and the majority had an education level at college/university and above (63.9%). 

### 3.2. Socio-Demographic Characteristics of the ASD Children 

The mean (SD) age of the ASD children was 7.45 (3.54) years. The majority were boys (82.8%), with co-morbidity (50.2%), and were registered with the Social Welfare Department (58.6%). The summary of the findings is presented in Table 2.

### 3.3. The Mean and Distribution of Responses for Each Question in Malay Version of PSS-10

Table 3 presents the descriptive statistics of perceived stress of the caregivers. Most of the participants responded in the “sometimes” category for the majority of the 10 items in the scale (37.4% to 61.7%). The majority of caregivers (61.7%) said that they sometimes had been upset because of something that happened unexpectedly. A good number of caregivers (10.2%) fairly often felt that difficulties were piling up so high that they could not overcome them. The mean (SD) total PSS-10 score of all 227 caregivers was 20.84 (4.72), with a minimum score of 1.00 and maximum score of 30.00. According to PSS-10 scoring by Kelly and Percival [22], the mean total perceived stress score in the current study was considered much higher than average and health concern was also high (total score more than 20). The interpretations of perceived stress levels according to total PSS-10 score are displayed in Table 4.

### 3.4. The Predictors of Perceived Stress among Caregivers with ASD Children

In attempting to understand the factors that could predict perceived stress among caregivers with ASD children, all the variables in the study and the Malay version of the PSS score were entered into regression analysis, as these factors were significantly associated with caregivers’ stress. There were three significant predictors of perceived stress among caregivers of children with ASD, a summary of which is displayed in Table 5.

Final model equation of perceived stress among caregivers with ASD children: 26.34 + (1.76 × distance from residence to tertiary care, 25 km or more) + (3.06 × transportation constraint to child treatment center) + (2.41 × caregiver concern of LD present in their child).

## 4. Discussion

This study aimed to determine the level of perceived stress among caregivers with an ASD child and stress predictors.

### 4.1. Level of Perceived Stress among Caregivers of Children with ASD according to PSS-10

In the current study, the mean total score of PSS-10 was 20.84, indicating that the stress level was much higher than an average score of between 12 and 15 [22]. The current finding is consistent with previous studies that found parents of individuals with ASD were under a considerable stress [8,10,11,23]. The studies conducted by Schieve and Blumberg [24] and Freedman and Kalb [25] support the findings by stating that caregivers of children with ASD were also more likely to have high levels of stress compared to caregivers with other children with disabilities. The perceived stress level in the current study was determined according to the PSS-10 scoring interpretation by Kelly and Percival [22]. 

It is quite worrying when 10.2% of the caregivers fairly often felt that difficulties were piling up so high that they could not overcome their problems. This is consistent with the findings by Kline [26]. The higher stress levels in caregivers might affect the adjustment to taking care of their child with ASD and increase susceptibility to stress-induced illness leading to higher mortality risk [27].

Nevertheless, a study revealed that most Malay caregivers use religious belief as their coping method, in addition to acceptance, optimist attitude, and active coping [28], even though the facilities and services available to disabled people in this country are relatively limited [29].

### 4.2. Predictors of Perceived Stress among Caregivers with ASD Children

The current study has revealed three interesting and significant predictors of perceived stress among caregivers of children with ASD. A positive relationship was observed with the PSS score for the distance of 25 km or more from the patient’s residence to the tertiary care center and for having difficulties with transportation to tertiary care. The caregivers who perceived stress with the distance involved showed an increment in PSS score by 1.76. In the current study, the distance from their residence to the healthcare facility was self-reported by the caregivers, which is not necessarily related to a lack of available free time for parenting, housing conditions, and socioeconomic status. To ensure that the distance reported in the questionnaire was accurate, we double-checked the distance using Google Maps. Whereby, the caregivers who perceived stress with a transportation constraint to tertiary care showed an increment in PSS score by 3.06. Caregivers living in under-resourced or more distant areas from the diagnosis center could attribute these issues to delaying or missing the child’s intervention program [30,31]. Consequently, the child’s condition, cognitive functioning, social interaction, and ASD symptoms were not able to be improved and could have even worsened. By not being able to be present during follow-up at tertiary care, caregivers were not provided with available support services that could help them and their child. This situation eventually intensified the level of their perceived stress. In tackling this issue, psychosocial interventions also need to be provided during primary care and secondary care. Having a therapist visit the community clinic or community rehabilitation center located near the caregivers’ residence may solve the problem of therapy compliance. Ultimately, this may also improve the child’s symptoms and reduce the caregivers’ stress level and improve quality of life [32]. Besides, healthcare professionals should always keep in touch and follow up with families who are lacking in resources and experiencing a pile up of stressors so that they are able to address the caregivers’ actual needs and desires.

Psychiatric comorbidities, such as LD, are common among children with ASD [2]. The current study showed an increment of PSS score by 2.36 among caregivers whose children have an LD. The finding is in line with Kline [26] and Freedman and Kalb [25] in which caregivers encounter heightened stress and other inter and intra personal difficulties when raising ASD children with comorbidities. In order to improve the caregivers’ stress level, healthcare professionals should offer assistance. The consultation should not focus only on the child’s specific diagnosis but also include an assessment of the caregivers’ stress. Issues pertaining to other family stressors also need to be included in ASD management. Specific and validated tools should be used that cover all aspects of family domains, such as family vulnerability, views and appraisals of the illness or disability, resources and supports, and the ability to cope and solve problems. 

In addition, caregivers should be part of a social or parent support group. Parent support groups work because multifamily teams create a meaningful context of exchange to help caregivers reduce social isolation and allow them to access information about their child especially in the management of ASD. Parents can learn advocacy skills, gain confidence, and practice problem solving and coping strategies in their caregiving role [11,33]. Programs that include parent training may help to boost perceived competence and positively impact caregiver-perceived stress.

### 4.3. Study Limitations

The findings in this study were based on caregivers’ self-reporting. As such, inaccurate reporting might have biased our findings. The majority of the sample were from caregivers attending the intervention at government healthcare facilities. We did not include caregivers from private centers and those who defaulted in the intervention program. This was due to the difficulty in obtaining a proper list from the private centers. Without a comparison group, it is difficult to justify that certain factors, for example, owning a car or proximity to healthcare facilities, are specific to ASD. Further study with different groups of disabilities is required to compare the influence of these factors on caregivers’ stress. 

The current study also did not look into the severity of ASD or the genetic component of ASD that aggravated stress levels. The current study was conducted in Kelantan, which consists of 96.0% Malay residents [34]. Thus, we could not generalize the current findings. We were also not able to speculate whether caregivers with less stress were likely to participate. However, because our sampling method was a stratified random sampling, we assumed that regardless of stress level, caregivers were included in our sample. We also believed that those who refused to respond did so only due to work commitments. Despite these limitations, this study provides a valuable picture of vulnerable families in Kelantan who need proper care for their ASD children.

### 4.4. Future Research

Many paths can be taken by caregivers who have a child with ASD to cope with parental stress. Recognizing the benefits and limitations of this study allowed us to understand that the variables among caregivers are an important area of research, even when the primary client receiving treatment is the child. A start may be to extend the current study to explore in depth the significant factors in our culture that contribute to perceived stress among caregivers of children with ASD. It is also recommended that future study would benefit a larger sample to enhance external validity. The current study has contributed to the available literature by understanding caregivers’ experiences in raising their child with ASD. We hope these findings will provide a positive impact and offer recommendations to improve caregiver stress.

## 5. Conclusions

In conclusion, caregivers of children with ASD in this population had much higher levels of stress than the average, as recommended by Kelly and Percival [22]. Higher perceived stress was significantly predicted based on the distance from the caregivers’ residence to tertiary care of 25 km or more, having a problem with transportation to the child’s treatment center, and the presence of learning disabilities.

## Figures and Tables

**Table 1:** Socio-demographic characteristics of the caregivers (*n* = 227).

Variables	Frequency (%)	Mean (SD)
Age		38.91 (8.26)
Occupation		
Professional	89 (39.2)	
Non-professional	59 (26.0)	
Housewife/unemployed	79 (34.8)	
Main caregiver		
Mother	127 (55.9)	
Father	19 (8.4)	
Both	73 (32.2)	
Others	8 (3.5)	
Caregiver education		
Secondary school and below	82 (36.1)	
College/university and above	145 (63.9)	
Household income (USD)		
≤USD 490	63 (27.8)	
>USD 490 to <USD 1225	88 (38.8)	
USD 1225 to <USD 1960	41 (18.1)	
≥USD 1960	35 (15.4)	
Problem with transportation		
Yes	26 (11.5)	
No	201 (88.5)	
Has a medical issue		
Yes	11 (4.8)	
No	216 (95.2)	

SD: Standard deviation.

**Table 2:** Socio-demographic characteristics of the autism spectrum disorder (ASD) children (*n* = 227).

Variables	Frequency (%)	Mean (SD)
Age		7.45 (3.54)
Gender		
Boy	188 (82.8)	
Girl	39 (17.20)	
ASD with comorbidity		
Yes	114 (50.2)	
No	113 (49.8)	
Age of child when caregiver started showing concern (year)		2.66 (1.66)
Caregiver concern and worries when the child has:		
Speech delay		
Yes	212 (93.4)	
No	15 (6.6)	
Delayed walking		
Yes	46 (20.3)	
No	181 (79.7)	
Social problems		
Yes	137 (60.4)	
No	90 (39.6)	
Dislikes change		
Yes	84 (37.0)	
No	143 (63.0)	
Hyperactive child		
Yes	112 (49.3)	
No	115 (56.8)	
Learning disability		
Yes	98 (43.2)	
No	129(56.8)	
Medical problems		
Yes	19 (8.4)	
No	208 (91.6)	
Hearing difficulty		
Yes	34 (15.0)	
No	195 (85.0)	
Hypersensitivity		
Yes	84 (37.0)	
No	143 (63.0)	
Sleeping problems		
Yes	77 (33.9)	
No	150 (66.1)	
Does the child attend school?		
No schooling	43 (18.9)	
Government school	67 (29.5)	
Private school	117 (51.6)	
Age of child at diagnosis of ASD (year)		4.98 (1.85)
Age of child when caregiver sought help for the first time (year)		3.72 (2.08)

**Table 3:** The mean and distribution of responses for each question in the Malay version of Perceived Stress Scale-10 Items (PSS-10) (*n* = 227).

Statements	Mean (SD)	Frequency (%)					Min-Max
		Never	Almost Never	Sometimes	Fairly Often	Very Often	
1. Dalam tempoh sebulan ini, berapa kerap anda marah disebabkan sesuatu itu berlaku tanpa anda jangka? *In the last month, how often have you been upset because of something that happened unexpectedly?*	2.04 (0.78)	14 (6.2)	20 (8.8)	140 (61.7)	50 (22.0)	3 (1.3)	0, 4
2. Dalam tempoh sebulan ini, berapa kerap anda merasakan bahawa anda tidak boleh mengawal sesuatu perkara yang penting dalam hidup anda? *In the last month, how often have you felt that you were unable to control the important things in your life?*	1.72 (0.89)	26 (11.5)	51 (22.5)	113 (49.8)	35 (15.4)	2 (0.9)	0, 4
3. Dalam tempoh sebulan ini, berapa kerap anda berasa gementar dan tertekan? *In the last month, how often have you felt nervous and “stressed”?*	1.70 (0.98)	30 (13.2)	54 (23.8)	105 (46.3)	30 (13.2)	8 (3.5)	0, 4
4. Dalam tempoh sebulan ini, berapa kerap anda berasa yakin dengan kebolehan anda untuk mengurus masalah peribadi anda? *In the last month, how often have you felt confident about your ability to handle your personal problems?*	2.43 (0.78)	11 (4.8)	-	103 (45.4)	106 (46.7)	7 (3.1)	0, 4
5. Dalam tempoh sebulan ini, berapa kerap anda berasa bahawa perkara yang berlaku mengikut apa yang anda rancangkan? *In the last month, how often have you felt that things were going your way?*	2.62 (0.65)	3 (1.3)	1 (0.4)	87 (38.3)	125 (55.1)	11 (4.8)	0, 4
6. Dalam tempoh sebulan ini, berapa kerap anda dapati bahawa anda tidak boleh mengawal perasaan dengan semua perkara yang telah anda lakukan? *In the last month, how often have you found that you could not cope with all the things that you had to do?*	1.72 (0.81)	19 (8.4)	55 (24.2)	125 (55.1)	26 (11.5)	2 (0.9)	0, 4
7. Dalam tempoh sebulan ini, berapa kerap anda telah dapat mengawal ketidak selesaan dalam hidup anda? *In the last month, how often have you been able to control irritations in your life?*	2.64 (0.69)	4 (1.8)	-	85 (37.4)	123 (54.2)	15 (6.6)	0, 4
8. Dalam tempoh sebulan ini, berapa kerap anda berasa bahawa anda berjaya mengatasi semua masalah? *In the last month, how often have you felt that you were on top of things?*	2.56 (0.68)	6 (2.6)	-	89 (39.2)	126 (55.5)	6 (2.6)	0, 4
9. Dalam tempoh sebulan ini, berapa kerap anda telah marah disebabkan perkara yang berlaku di luar kawalan anda? *In the last month, how often have you been angered because of things that were outside of your control?*	1.94 (0.83)	15 (6.6)	39 (17.2)	120 (52.9)	51 (22.5)	2 (0.9)	0, 4
10. Dalam tempoh sebulan ini, berapa kerap anda berasa kesusahan yang melampau sehingga anda tidak dapat mengatasinya? *In the last month, how often have you felt that difficulties were piling up so high that you could not overcome them?*	1.52 (0.93)	39 (17.2)	59 (26.0)	106 (46.7)	19 (8.4)	4 (1.8)	0, 4
Total score	20.87 (4.72)						

**Table 4:** Perceived stress and health concerns according to score.

Total Score	Perceived Stress Level	Health Concerns
0–7	Much lower than average	Very low
8–11	Slightly lower than average	Low
12–15	Average	Average
16–20	Slightly higher than average	High
>20	Much higher than average	Very high

Source: Kelly and Percival [22].

**Table 5:** Predictors of perceived stress among caregivers with ASD children (*n* = 227).

Variables	Simple Linear Regression		Multiple Linear Regression	
	^a^ (95% CI)	*p*-Value	^b^ (95% CI)	*p*-Value
Distance from residence to tertiary care (km)				
Less than 25 km	0			
25 km or more	1.85 (0.21, 3.48)	0.031	1.76 (0.18, 3.34)	0.03
Having problems with transportation to child treatment center				
No	0			
Yes	2.49 (−0.04, 5.03)	0.055	3.06 (0.53, 5.61)	0.018
Caregiver concern of learning disability present in child				
No	0			
Yes	2.36 (0.75, 3.98)	0.004	2.42 (0.85, 3.98)	0.002

^a^ Crude regression coefficient. ^b^ Adjusted regression coefficient. Forward multiple linear regression method applied. Model assumption is fulfilled. There were no interactions among independent variables. No multi-collinearity was detected. Coefficient of determinants, *R^2^* = 10.0%.
